# Effect of Indian Ocean–Pacific SST Pattern in Autumn on Winter Wheat Climatic Yield in the North China Plain in the Following Year and a Possible Mechanism

**DOI:** 10.1038/s41598-019-55483-2

**Published:** 2019-12-12

**Authors:** Qingyan Xie, Jianping Li

**Affiliations:** 1 College of Global Change and Earth System Sciences (GCESS), Beijing Normal University, Beijing, 100875 China; 2 Key Laboratory of Physical Oceanography–Institute for Advanced Ocean Studies, Ocean University of China and Qingdao National Laboratory for Marine Science and Technology, Qingdao, 266100 China

**Keywords:** Climate-change impacts, Environmental impact

## Abstract

Ensuring stable crop yield increases to meet rising demand is an important issue globally, particularly when accounting for climate change. In this study, using observations, reanalysis datasets, and the Hodrick and Prescott filter method, we find that changes in a distinct pattern of Indian Ocean–Pacific five-pole (IPFP) SST (sea surface temperature) are strongly linked to the ensuing year’s winter wheat climatic yield (the part of yield that fluctuation caused by climatic factors change) in the North China Plain (NCP), which is the main production region of winter wheat in China. Here we define a normalized IPFP index (IPFPI) and demonstrate that the autumn IPFPI (1948–2014) is well correlated with the ensuing year’s winter wheat climatic yield (1949–2015), particularly for October (*r* = 0.69; *n* = 67; *P* < 0.001). A composite analysis shows that the October IPFP is correlated with sowing-period and emergence-period climate factors in the NCP. When the October IPFP is in a positive phase, the atmosphere geopotential height fields and water vapor flux are bebefitial to rainfall formation in NCP, and the precipitation and soil moisture are higher in NCP and benefit winter wheat growth, thus increasing the climatic yield. In addition, accumulated rainfall and soil water content might influence winter wheat growth from sowing and emergence (autumn) to the returning green stage (following spring).

## Introduction

China produces more crops, and in particular more wheat, than any country in the world. It does so with only 7% of the earth’s arable land available to feed 20% of the world’s population^1^. According to the National Bureau of Statistics of China, in the past decade (2008–2017), China had harvested average 608.6 million tons of grain every year, of which 123.8 million tons were wheat, accounting for 20%. In the same period, the average yield of wheat was 708.5 million tons in the world every year, which China accounting for 17.5%. Thus, China plays an important role in wheat production and food security in the world. However, because of global warming, agriculture security and crop production are facing important risks and challenges that might affect quality of life and global stability^2^. In addition, China is a major contributor to global trade, and thus the effects of climate change in China will likely affect other nations through international food trade^3^. The North China Plain (NCP) is the primary growing region for winter wheat (Triticum aestivum L.) in China^4^. Shanxi, Hebei, Shandong, Henan, and Anhui provinces’ total sowing area and production were 65% and 73%, respectively, of the national values in 2016. In recent decades, the NCP has experienced a significant temperature increase and precipitation decrease^1,5^. These changes might influence winter wheat planting, growth, and yield^6^. Therefore, an investigation of changes in winter wheat yield in the NCP has important scientific and practical value, particularly in light of global climate change and population increases.

Many studies have reported that regional climatic factors during various growth periods affect winter wheat yield in the NCP^2,7,8^. These regional climatic factors include temperature^9^, precipitation^10^, solar radiation^6^ and soil moisture^11,12^, among others, and have impacts from seed sowing to harvest^4,13,14^. The IPCC has reported that temperature increases and rainfall decreases in a worst-case scenario may lead to Chinese wheat yield decreases of 20–36% over the next 20 to 80 years^15^. Tao *et al*.^16^ found that because of climate change (particularly warming during the growth season), China’s wheat yield may drop by 120 kilotons per decade. At regional scales, with the exception of Henan province, the impacts of temperature increases on wheat yield in all NCP provinces are negative. In addition, Zhuang *et al*.^17^ reported that the primary winter wheat growing region in North China is suffering from severe water shortages and irrigation deficiencies. Thus, drought plays an important role in NCP winter wheat yield. Adding complexity to the situation, the same climatic factors during the various development phases of winter wheat have different effects on yield. For example, in the seeding and emergence stages in the NCP, winter wheat yield is expected to decrease as temperature increases, and solar radiation has a negative correlation with winter wheat yield during these periods^17–19^. However, in the tillering, over-winter and heading stages, a positive correlation between temperature and winter wheat yield has been found in the NCP^17^. Precipitation is critical to winter wheat production from seeding and emergence to the re-greening stage. This period covers autumn, winter, and early spring, which is the dry season in the NCP. Thus, Chinese farmers often say that “rain in spring is as rare as oil”^17^. Many researchers have focused on how local climatic factors affect winter wheat yield in the NCP, but the factors that influence the local climate during the NCP winter wheat growing season have yet to be investigated.

Sea surface temperature (SST) is an important indicator of the climate system^20–22^. Changes in SST can affect the atmosphere through air–sea interactions^23^, and influence the climate of distant regions through several teleconnection patterns^24,25^, thus affecting regional agriculture development^26,27^ and crop production^28–30^. Cane *et al*.^31^ found that ENSO in 1994 led to changes in maize yield in Zimbabwe, and that NINO3 area SST is a good predictor of maize yield in this region. Woli *et al*.^32^ showed that the effect of ENSO on southern USA winter wheat yield varies with the winter wheat sowing date and the growing region latitude. In addition to ENSO effects, Capa-Morocho *et al*.^29^ found that tropical Pacific and Atlantic SST anomalies can cause North Atlantic Oscillation (NAO) changes, which together affect rainfall and temperature on the Iberian peninsula, influencing rain-fed wheat yield in this region. They suggested using early period SSTs to predict the wheat yield. After investigating how SST anomalies influence Canadian Prairie spring wheat yield, Hsieh *et al*.^26^ noted a negative–positive–negative SST triple teleconnection pattern in the North Pacific that had a significant relationship with West Canadian spring wheat yield during 1960–1997. The correlation coefficient between the triple SST index in March and the same year’s spring wheat yield in September reached 0.63 (P < 0.01). Thus, it is now possible to predict Canadian spring wheat yield half-a-year in advance. Travasso *et al*.^27^ studied crop yield in the Pampas region, Argentina’s main grain-producing region, and found that monthly SST anomalies in the equatorial Pacific and South Atlantic are well correlated with this region’s maize and wheat yield, particularly when using the SST signal before crop sowing.

In summary, multiple studies have described how remote SST anomalies are related to local climate factors and influence regional crop growth, development and production, and have characterized the relationship between SST signals and crop yield. However, only a few researchers have investigated the physical mechanisms that control how SST anomalies affect crop yield in distant regions and the role of local circulation and climate in these processes, particularly for winter wheat in the NCP. Therefore, an investigation of the interaction of “remote SST–local (NCP) circulation/climate–local crop (winter wheat) yield” is warranted. In this study, three key themes are addressed: (i) the link between NCP winter wheat climatic yield and remote SST signals, (ii) a mechanism describing how remote SST impacts future local climate factors in the NCP, and (iii) remote SST signals how to influence winter wheat climatic yield in the NCP.

## Results

### Relationship between autumn IPFP and NCP winter wheat climatic yield of the following year

**Figure 1:**
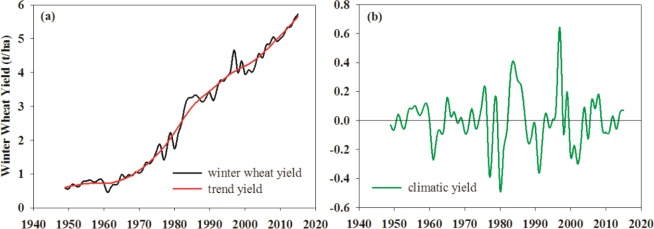
Time series of annual winter wheat yield (t/ha) in the NCP (1949–2015). (**a**) Original annual winter wheat yield (black solid line) and corresponding HP filter trend yield (red solid line); and (**b**) climatic yield of winter wheat determined using the HP filter.

Using the HP filter method, the NCP winter wheat climatic yield was computed for 1949 to 2015. Figure 1a shows the original annual winter wheat yield and the corresponding HP-filter trend yield. Figure 1b shows the climatic yield of winter wheat obtained from the HP filter. The NCP winter wheat yield has continued to increase over the past several decades, but the climatic yield fluctuates during the same period. The increasing trend yield can be attributed to improvements in areas such as breeding techniques, field management, irrigation, and fertilization. The fluctuating climatic yield might be caused by changes in climatic factors.

**Figure 2:**
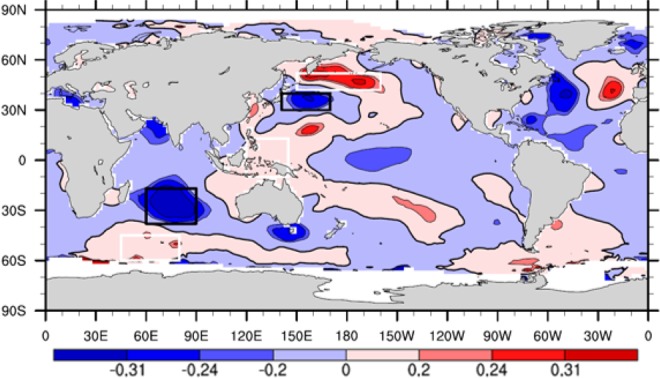
Correlation coefficients between winter wheat climatic yield in the NCP during 1949−2015 and global SST during the previous October (1948−2014). Three positive-correlation areas (white boxes) and two negative-correlation areas (black boxes) were chosen to construct the IPFPI. The correlation coefficients of ±0.2, ±0.24, and ±0.31 are statistically significant at the 90%, 95%, and 99% levels, respectively (Student’s *t-*test), (map produced by NCL Version 6.6.2, http://dx.doi.org/10.5065/D6WD3XH5)^46^.

Because NCP winter wheat is harvested each year in June, we computed the correlation coefficients of winter wheat climatic yield and global monthly SST one year prior to the winter wheat harvest. Thus, monthly SST data from July of the previous year to June of the harvest year were used in the correlation analysis with winter wheat climatic yield. We found that from July to December (because October has the strongest signal, we focus on results from October) of the year prior to the harvest, there were five highly correlated centers following the pattern “positive–negative–positive–negative–positive” from the southern Indian Ocean through the Indo-Pacific Warm Pool to the northwest Pacific (Fig. 2).

According to the domain and pattern of the highly correlated regions, the normalized SST sequence was used to define an Indian Ocean–Pacific SST five-pole index (IPFPI):

1 $${\rm{IPFPI}}=({\rm{A1}}\ast +{\rm{A2}}\ast +{\rm{A3}}\,\ast )/6\mbox{--}({\rm{B1}}\ast +{\rm{B2}}\,\ast )/4,$$

where *A1*, *A2*, *A3*, *B1*, and *B2* denote the regional mean SST in (60°S–45°S, 45°E–80°E), (10°S–13°N, 120°E–145°E), (42°N–52°N, 150°E–160°W), (38°S–17°S, 60°E–90°E), and (30°N–40°N, 141°E–170°E), respectively. An asterisk indicates a normalized value.

**Figure 3:**
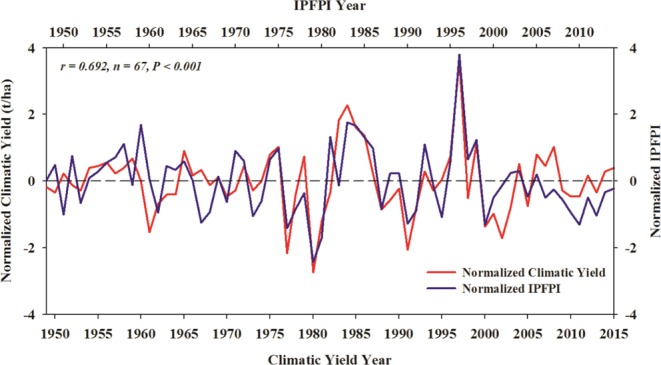
Time series of normalized winter wheat climatic yield in the NCP during 1949–2015 (red; lower abscissa) and normalized IPFPI during the previous October (1948–2014; blue; upper abscissa), and their correlation coefficient (left upper corner; statistically significant at the 99.9% level).

The correlation coefficient between the normalized IPFPI during autumn of 1948–2014 and the normalized winter wheat climatic yield in the NCP of the following year (1949–2015) is 0.69 (*n* = 67; Fig. 3), indicating a significant positive correlation (*P* < 0.001). However, the relationship captured by the IPFPI between remote SST signals and local NCP atmospheric circulations, and the effects on local climate factors during key periods of winter wheat growth (the seeding and emergence stages) as well as the overall impact on winter wheat production, require further analysis.

### Relationships between local NCP climatic factors, the IPFP of the same period, and winter wheat climatic yield

Temperature and precipitation are the main climatic factors influencing crop growth, development and yield fluctuations. Suitable temperatures and abundant rainfall are necessary conditions for normal crop grow and high yield. In addition, soil moisture affects crop yield, particularly in rain-fed agricultural areas.

**Figure 4:**
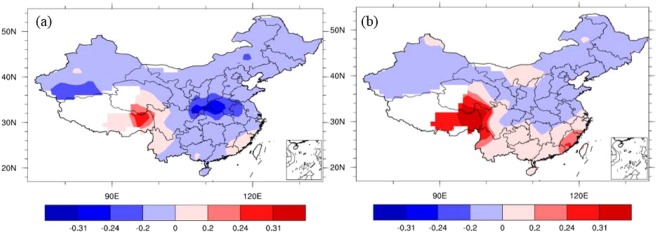
Correlation coefficients between October temperatures (160 stations’ data), IPFPI, and winter wheat climatic yield. (**a**) Correlation coefficients between the October temperature in China and the IPFPI during 1951−2014; and (**b**) correlation coefficients between the temperature in October during 1951−2014 and winter wheat climatic yield in the NCP during the following year (1952−2015). The correlation coefficients of ±0.2, ±0.24, and ±0.31 are statistically significant at the 90%, 95%, and 99% levels, respectively (Student’s *t-*test), (maps produced by NCL Version 6.6.2, http://dx.doi.org/10.5065/D6WD3XH5)^46^.

Figure 4a shows that the local NCP temperature in October has a significant negative correlation with the October IPFPI during 1951–2014. This suggests that the IPFP negatively impacts the NCP local temperature in October, which is the sowing period for winter wheat in the NCP. Figure 4b shows that the temperature in October during 1951–2014 has a weak negative influence on the corresponding winter wheat climatic yield in NCP in the following year (1952–2015). This suggests that temperature fluctuations in the sowing period have a negative impact on the following year’s winter wheat climatic yield. These findings are similar to those of others studies. Zhuang *et al*.^17^ found that in northern China, higher temperatures during the seeding and emergence stages result in a reduced yield of winter wheat. The rate of yield decrease was about –40 kg ha^−1^ °C^−1^. On a countrywide scale, rising temperatures might have contributed to a 4.5% reduction in wheat yield during 1979–2002^18^, and warmer daytime temperatures were likely to have decreased wheat yield by 6%–20% per 1 °C of temperature increase^16^. These results indicate that winter wheat yield are negatively affected by warming conditions. This may be because winter wheat is a chimonophilous crop (i.e., it prefers a cool climate). If temperatures increase, particularly during key growth stages such as the seeding and emergence periods, winter wheat growth might accelerate and advance the phenological period to before winter. This could expose winter wheat to low temperatures, wind, and drought during winter and the following spring.

**Figure 5:**
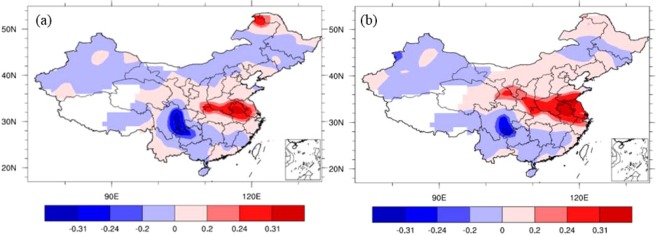
Correlation coefficients between October precipitation (160 stations’ data), IPFPI, and winter wheat climatic yield. (**a**) Correlation coefficients between October precipitation and IPFPI during 1951−2014; and (**b**) correlation coefficients between October precipitation in China during 1951−2014 and winter wheat climatic yield in the NCP during the following year (1952−2015). The correlation coefficients of ±0.2, ±0.24, and ±0.31 are statistically significant at the 90%, 95%, and 99% levels, respectively (Student’s *t-*test), (maps produced by NCL Version 6.6.2, http://dx.doi.org/10.5065/D6WD3XH5)^46^.

**Figure 6:**
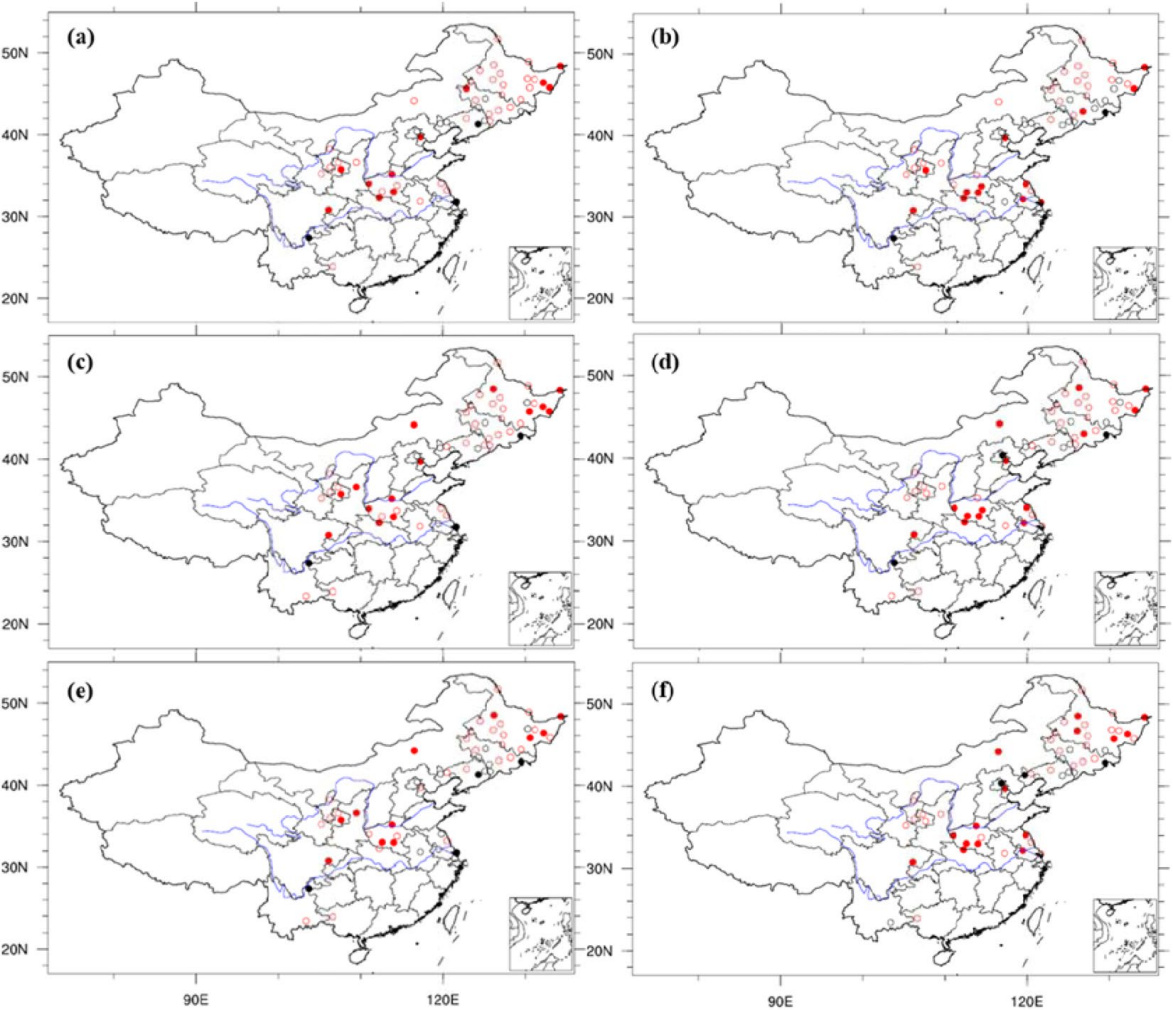
Correlation coefficients between October soil moisture at various depths, IPFPI, and winter wheat climatic yield. (**a**), (**c**), and **(e**) Correlation coefficients between 10-, 20-, and 30-cm soil moisture during October in China and IPFPI for 1982−2010, respectively; and (**b**), (**d**), and (**f**) correlation coefficients between 10-, 20-, and 30-cm soil moisture during October in China for 1982−2010 and winter wheat climatic yield in the NCP during the following year (1983−2011), respectively. Red dots indicate positive correlations and black dots indicate negative correlations. Open circles indicate no statistically significant correlation, and solid circles indicate a statistically significant correlation at the 90% level, (maps produced by NCL Version 6.6.2, http://dx.doi.org/10.5065/D6WD3XH5)^46^.

In contrast to the temperature effects, Fig. 5a shows that the local NCP precipitation in October has a significant positive correlation with the October IPFPI during 1951–2014, and the results similar with 0.5 × 0.5° grid data’s results (we don’t show the figures here). This suggests that the IPFP has a positive impact on local NCP precipitation in October. Figure 5b shows that precipitation in October during 1951–2014 had a significant positive influence on the ensuing year’s winter wheat climatic yield in the NCP (1952–2015). This suggests that increased precipitation during the sowing and emergence periods leads to higher winter wheat climatic yield the following year. Soil moisture is closely related to precipitation. Soil moisture at various depths in October is positively correlated with the IPFPI of the same period in the NCP during 1982–2010 (Fig. 6a,c,e). This suggests that the IPFP increases soil moisture at various depths in October. Similarly, soil moisture at various depths in October during 1982–2010 is significantly positively correlated with the corresponding winter wheat climatic yield in the NCP of the following year (1983–2011; Fig. 6b,d,f). This suggests that during the sowing and emergence periods, soil moisture has a significant positive stimulating effect on winter wheat growth, development, and climatic yield.

Drought results in reduced crop yield in semi-arid to semi-humid areas^33–35^, particularly for a rain-fed agricultural system^36–38^^,^. Before and after October corresponds to the winter wheat seeding and emergence periods in the NCP^7,10^. Precipitation and soil moisture at these stages are important to winter wheat growth^4^. This applies not only to October, but also to the several months before the seeding period, as precipitation and soil moisture have continuous effects on winter wheat^39^. Because the NCP has a temperate monsoon climate, severe droughts occur, particularly in the winter half of the year (October to March). Thus, precipitation and soil moisture play an important role in winter wheat production from emergence to the turning-green and jointing stages. Hence, our results demonstrate that water supply conditions during the vegetative growth phase are closely linked to winter wheat growth and yield, in agreement with previous work^5,17^.

### Possible mechanism of IPFP influence on local NCP climatic factors

**Figure 7:**
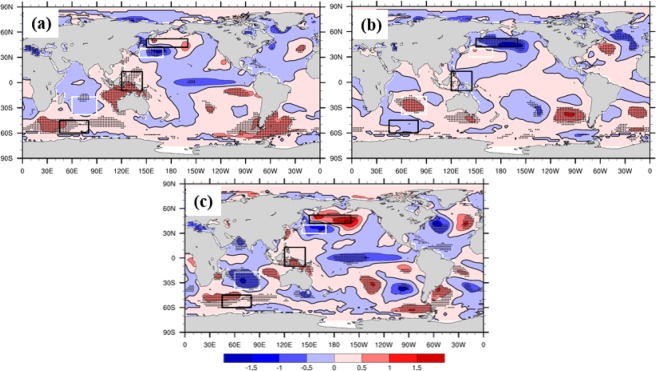
Composite global October SST anomalies (°C). (**a**) Highest IPFPI years, (**b**) lowest IPFPI years, and (**c**) differences between the highest and lowest IPFPI years. Anomalies that are statistically significant at the 90% level (Student’s *t*-test) are stippled. The highest and lowest IPFPI are selected according to Table 1, (maps produced by NCL Version 6.6.2, http://dx.doi.org/10.5065/D6WD3XH5)^46^.

In this study, composite analysis was used to investigate how the IPFP affects local NCP climatic factors. The five highest and five lowest October IPFPI values along with the corresponding winter wheat climatic yield of the following year were selected for the analysis (Table 1). A significant difference exists between the averages of the highest and lowest October IPFPI values, and a similar difference exists between the average climatic yield (Table 1). Figure 7a,b shows that the composite SST anomalies of the five highest (and lowest) October IPFPIs are related to a positive (and negative) phase of the five cells of the India Ocean–Pacific SST. The composite difference also reflects the significant difference between the IPFP positive and negative phases (Fig. 7c). Thus, different IPFP phases correspond to different SST anomaly patterns. This may lead to different atmospheric cycling patterns through air–sea interactions and teleconnections that impact local NCP climate and circulation.

**Table 1:** Composite analysis of the highest and lowest October IPFPI years and corresponding winter wheat climatic yield in the NCP in the following year.

Highest October IPFPI years	1983	1984	1985	1996	1998	Average
October IPFPI	0.65	0.61	0.48	1.39	0.45	0.716*
Corresponding winter wheat climatic yield (t/ha) in the NCP in the following year	0.4	0.28	0.24	0.64	0.2	0.352*
Lowest October IPFPI years	1976	1979	1980	1990	1999	**Average**
October IPFPI	−0.52	−0.89	−0.62	−0.47	−0.48	−0.596*
Corresponding winter wheat climatic yield (t/ha) in the NCP in the following year	−0.38	−0.48	−0.21	−0.36	−0.24	−0.334*

*Stastically significant at the 99.9% level.

**Figure 8:**
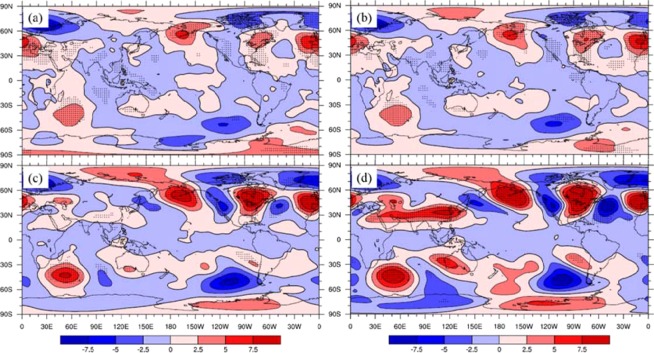
As in Fig. 7(c), but for geopotential height (10 gpm) fields in October. (**a**) 1000 hPa, (**b**) 850 hPa, (**c**) 500 hPa and (**d**) 200 hPa, (maps produced by NCL Version 6.6.2, http://dx.doi.org/10.5065/D6WD3XH5)^46^.

The different geopotential height fields during the highest and lowest IPFPI years were selected for composite difference analysis. The results of composite difference showed that 1000 hPa (Fig. 8a) and 850 hPa (Fig. 8b) in NCP showed negative anomalies in geopotential height, while positive anomalies in geopotential height were observed at 500hPa (Fig. 8c) and 200hPa (Fig. 8d), and statistically significant at 90% level. This showed that when IPFPI appears positive abnormal high value year, there were low pressure anomalies near the ground and low altitude atmosphere in NCP, while high pressure anomalies appear in the middle and upper atmosphere. This kind of high and low pressure configuration at vertical height was benefit for the updraft in NCP, which made the temperature of NCP at the lower level and the precipitation increased, which was beneficial to the growth and yield increase of winter wheat, and vice versa.

**Figure 9:**
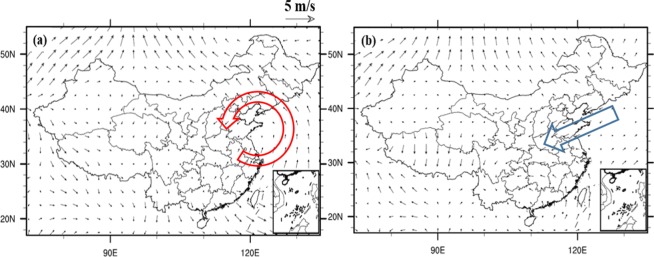
As in Fig. 7(a,b), but for October 700-hPa circulation anomalies (m/s), (maps produced by NCL Version 6.6.2, http://dx.doi.org/10.5065/D6WD3XH5)^46^.

**Figure 10:**
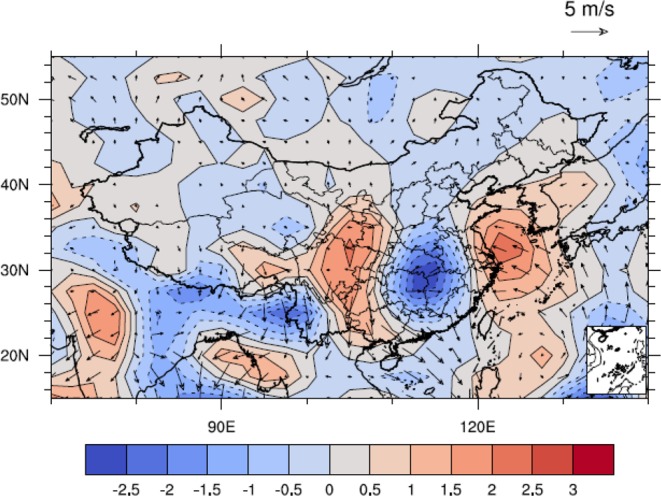
As in Fig. 7(c), but for October water vapor flux (g·cm^−1^·hPa^−1^·s^−1^) and water vapor flux convergence at 700 hPa, (maps produced by NCL Version 6.6.2, http://dx.doi.org/10.5065/D6WD3XH5)^46^.

**Figure 11:**
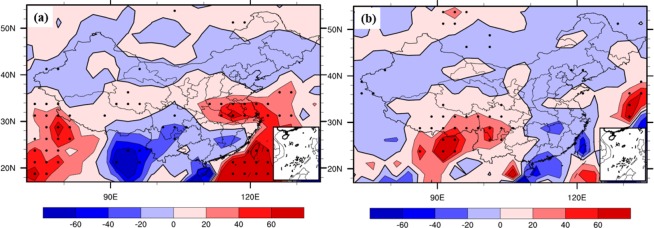
As in Fig. 7(a,b), but for October GPCP precipitation anomalies (mm), (maps produced by NCL Version 6.6.2, http://dx.doi.org/10.5065/D6WD3XH5)^46^.

**Figure 12:**
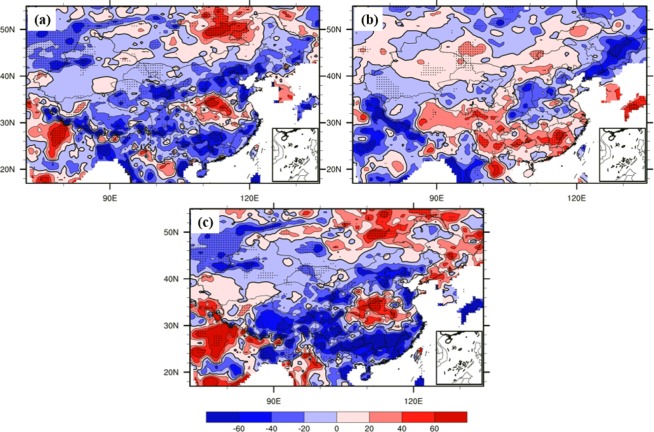
As in Fig. 7, but for October soil moisture anomalies (mm), (maps produced by NCL Version 6.6.2, http://dx.doi.org/10.5065/D6WD3XH5)^46^.

Because of the existence of different IPFP phases, different related circulation patterns might variously influence the NCP. Figure 9a shows that when the October IPFP is in a positive phase, it can cause a cyclone anomaly in the NCP at 700 hPa, which may decrease the air temperature and increase the precipitation in the winter wheat sowing and emergence periods. The composite differences of October water vapor flux and water vapor flux convergence at 700 hPa between highest and lowest IPFPI years have been showed (Fig. 10). We can find that the moisture come from the western Pacific Ocean transfer into and convergence in NCP, and may benefit for increase precipitation in this area. Figure 11a suggests a significant rainfall increase in the NCP when the IPFP is in a positive phase, consistent with the cyclone anomaly at 700 hPa in the NCP. However, when the October IPFP is in a negative phase, an eastern wind anomaly in the NCP exists at 700 hPa (Fig. 9b). Thus, the airflow does not converge in this area, which suppresses precipitation formation. Figure 11b shows a NCP precipitation decrease when the IPFP is in a negative phase in October. The October soil moisture is similar to the precipitation in the NCP. When the IPFP is in a positive phase, the NCP soil moisture anomaly shows a marked increase (Fig. 12a), and the NCP soil moisture anomaly decreases during negative IPFP phases (Fig. 12b), as also revealed in the composite difference (Fig. 12c).

These results indicate that the October IPFP could, through ocean–atmosphere interactions and atmospheric bridge effects^40^, impact the same-period local circulation in the NCP and further influence the temperature, precipitation, and soil moisture. Because of the high correlation between precipitation and soil moisture, when the IPFP is in a positive (negative) phase, precipitation and soil moisture anomalies increase (decrease) in the NCP during the winter wheat seeding and emergence stages, with important effects on subsequent winter wheat growth, leading to increased (decreased) yield. However, how autumn IPFP anomalies impact winter wheat growth remains to be determined.

**Figure 13:**
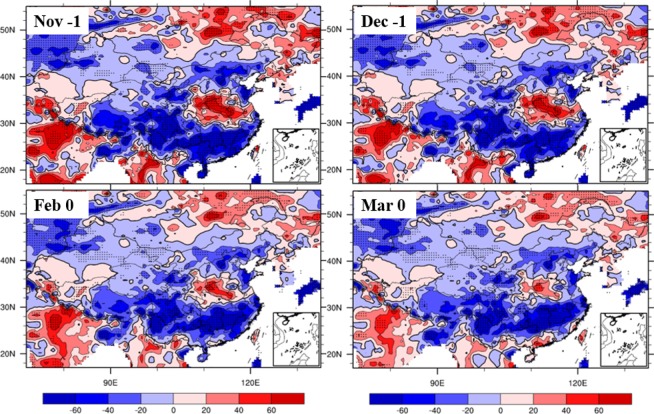
Composite differences in soil moisture (mm) between the highest and lowest IPFPI years from after sowing and emergence to the re-greening stage (November to March; data for January are omitted), (maps produced by NCL Version 6.6.2, http://dx.doi.org/10.5065/D6WD3XH5)^46^.

To investigate the continuous effects of autumn IPFP, precipitation, and soil moisture, we examined the NCP soil moisture from after the sowing stage to the re-greening stage in the highest-IPFP years. Figure 13 shows that in years with strong precipitation anomalies before and during the sowing stage, the NCP soil retains a high water content from November (after sowing and emergence) to March (re-greening stage). This indicates that with high precipitation, the soil stores more water to support winter wheat growth and development for a long period. This is important for winter wheat development because this period corresponds to the dry season in the NCP, when severe cold and dry winter monsoons can lead to low temperatures and little rainfall. However, higher water content in the soil could play a role in retaining heat and moisture, thus helping winter wheat successfully over-winter and increasing yield in summer.

## Discussion and Conclusion

We used a HP filter to calculate the North China Plain (NCP) winter wheat climatic yield during 1949−2015. The yield is significantly positively correlated with a pattern of Indian Ocean–Pacific five-pole (IPFP) SST of the previous autumn during 1948−2014. Over the past 67 years, their correlation coefficient is 0.69, significant at the 99.9% confidence level (two-tailed *t*-test). Therefore, we conclude that a close link exists between the IPFP in autumn and winter wheat climatic yield in the NCP in the following year.

We also found that the autumn IPFP has a significant relationship with the atmosphere signals (geopotential height fields), and local NCP climate factors in the same period (negative correlation with temperature, positive correlation with precipitation and soil moisture). We further explain how the autumn IPFP could impact local NCP circulation through ocean–atmosphere interactions and atmospheric bridge effects^40^. We found that the atmospheric geopotential fields at different heights from 1000 hPa to 500 hPa, exhibit a five-pole distribution similar to the SST field. This result showed that when there was an abnormal change in the IPFP, the atmosphere also showed the corresponding changes and signals. At the same time, the correlation between the climatic yield of winter wheat in NCP and the different geopotential height fields in October of the previous year showed that there was a negative correlation between the low altitude atmosphere (925 hPa and 850 hPa) in NCP and a significant positive correlation in the middle atmosphere (700 hPa and 500 hPa) (Fig. R5), which indicated that the configuration of this atmospheric geopotential height was benefit for the updraft in NCP. Therefore, the temperature decreased, the precipitation and soil moisture increased during the sowing period of winter wheat, which was contribute to the growth and yield of winter wheat (we don’t show the figures here). Thus, the IPFP’s anomaly change could through impact atmospheric signals’ variation and further influence the temperature, precipitation and soil moisture in the winter wheat sowing and emergence periods.

The temperature and precipitation in autumn are important for winter wheat sowing and emergence in the NCP. Lower temperatures and higher rainfall benefit winter wheat production, in agreement with our results. Finally, soil moisture also plays a key role in this process. We found that autumn precipitation continuity effects can, through soil moisture storage, aid winter wheat growth and development from autumn to the following spring, leading to fluctuations in the yield of winter wheat.

We have identified a connection between winter wheat yield and prior remote SSTs, and we have explained how remote SST impacts local climate in the NCP and influences the winter wheat yield. These results will help us to better understand how prior remote SSTs impact regional agricultural crop yield, which is important for decision makers in planning and deploying agricultural policies, and for farmers in improving agricultural activities and optimizing planting structures. In addition, we have described a way to use remote SST signals from a specific period to predict crop yield in an agricultural region. Future work will include how to utilize the October IPFP to predict the ensuing year’s winter wheat climatic yield in the NCP.

## Data and Methods

### Observational dataset and statistical methods

**Figure 14:**
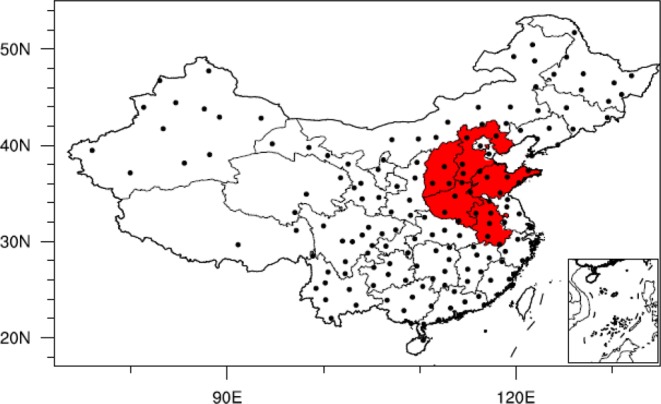
The location of North China Plain (red area, five provinces: Hebei, Shanxi, Shandong, Henan and Anhui) and the China 160 meteorological stations’ location (black solid dots) in this study, (maps produced by NCL Version 6.6.2, http://dx.doi.org/10.5065/D6WD3XH5)^46^.

This study analyses the NCP (including Shanxi, Hebei, Shandong, Henan and Anhui provinces) winter wheat yield from 1949 to 2015. Meteorological data include surface air temperature (SAT), ER-SST, precipitation, geopotential height (GPH), u/v wind and soil moisture from NOAA (https://www.esrl.noaa.gov/psd/data/gridded/data.ncep.reanalysis.pressure.html). To validate these data, monthly temperature and precipitation data from 160 Chinese stations from 1951 to 2015 are also used. The five provinces of NCP and 160 stations’ location showed in Fig. 14. Details of the datasets are provided in Table 2.

**Table 2:** Datasets used in this study.

Variables/Factors	Unit	Domain	Period	Resolution	Data Source
Wheat (Triticum aestivum)	t/ha	China	1949**–**2015	Provincial	Ministry of Agriculture and Rural Affairs of the People’s Republic of China
SAT	°C	Global	1948**–**2015	2.5° × 2.5° (Monthly)	NCEP/NCAR Reanalysis 1^47^
Precipitation	mm	Global	1979**–**2015	2.5° × 2.5° (Monthly)	GPCP^48^
Precipitation	mm	Global	1979**–**2015	2.5° × 2.5° (Monthly)	CMAP^49^
SST	°C	Global	1948**–**2015	2° × 2° (Monthly)	ER-SST V5^50^
Soil moisture	mm	Global land	1948**–**2015	0.5° × 0.5° (Monthly) Surface layer	CPC Soil Moisture^51,52^
Soil moisture	m^3^/m^3^	China	1982**–**2010	55 Meteorological Stations (Monthly, from April to October) 3 layers	^53,54^
GPH	gpm	Global	1948**–**2015	2.5° × 2.5° (Monthly) 17 layers	NCEP/NCAR Reanalysis 1^47^
*u* wind	m/s	Global	1948**–**2015	2.5° × 2.5° (Monthly) 17 layers	NCEP/NCAR Reanalysis 1^47^
*v* wind	m/s	Global	1948**–**2015	2.5° × 2.5° (Monthly) 17 layers	NCEP/NCAR Reanalysis 1^47^
SAT	°C	China	1951**–**2015	160 Meteorological Stations (Monthly)	China Meteorological Administration
Precipitation	mm	China	1951**–**2015	160 Meteorological Stations (Monthly)	China Meteorological Administration
Precipitation	mm	China	1961**–**2013	0.5° × 0.5° (Monthly)	China Meteorological Administration

We used two-tailed *t*-test to calculate statistical significance. This study also used other statistical methods, including a Hodrick and Prescott (HP) filter, correlation analysis, linear regression, and composite analysis, which are not described in detail here, except for the HP filter.

### Climatic yield

Actual crop yield is made up of three parts: the trend yield (that part due to economic and technical development, agricultural management, utilization of pesticides and fertilization, and other human factors), the climatic yield (that part due to changing climatic factors), and a random error term^17,41^. We quantify the yield as follows:

2 $$Y=Yt+Yc+\varepsilon ,$$

where *Y* is the actual crop yield, Yt is the trend yield, Yc is the climatic yield, and ε is the random error term (for a Gaussian distribution with an average value of zero). The separation of Yc from Y can be difficult. Unfortunately, no method can exactly extract Yc from Y, so we must apply some estimation of Yc. Various techniques have been used to estimate Yc, including linear trends, slip averaging (three or five point), quadric-form trends, exponential trends, and HP filters^17,26^. These methods each have advantages and disadvantages. Through a trial and error process, we decided to use the HP filter in this study, which is described in detail in the next section.

### HP Filter

The HP filter method is widely used to extract trends from time series. It was proposed by Hodrick and Prescott in the 1980s, and improved by Hodrick and Prescott^42^. It is a decomposition method that is applied to one-time series in state space. It can be applied to, for example, crop production that comprises a long-term trend and a short-term fluctuation^43^. The following three points describe a HP filter: (i) a HP filter is an algorithm for extracting a smooth curve from time series data; (ii) a HP filter can be regarded as a special projection designed to extract a signal gt from time series data Yn (here, Yn is a superposition of gt and orthogonal noise ct); and (iii) a HP filter can be regarded as a type of high-pass filter. The high-frequency part passes through the filter and the low-frequency part is removed. It can be used to isolate high-frequency components whose period is <8 years^44,45^.

It is generally believed that there are two components of grain yield: a low-frequency component and a high-frequency component. These components can be separated using a HP filter. Let the crop yield sequence be *Yt* (*t = 1, 2, 3, …, n*, where *n* is the sample size), which is time series data that includes a long-term trend (*gt*) and a short-term fluctuation (*ct*). The HP filter can be used to separate *Yt* as follows:

3 $$Yt=gt+ct,$$

where *gt* is defined as a solution to the following minimization problem:

4 $$\min \{{\mathop{\sum }\limits_{t=1}^{n}({y}_{t}-{g}_{t})}^{2}+\lambda {\mathop{\sum }\limits_{t=1}^{n}[({g}_{t+1}-{g}_{t})-({g}_{t}-{g}_{t-1})]}^{2}\}.$$

When solving Eq. (4) as part of a HP filter, the results depend on the choice of the parameter *λ*. When *λ* = 0, the trend sequence satisfying the minimization problem, *gt*, is the actual grain yield sequence *Yt*. However, gradually increasing *λ* tends to smooth the trend sequence. As *λ* → + ∞, the trend sequence will approach one line infinitely. The most controversial part of this method relates to the choice of λ. Using other researches’ experience^44^, we select the following values for λ: 100 (annual data), 1600 (seasonal data), and 14,400 (monthly data).

This study utilized the Eviews8 software for the HP filter treatment. Because crop yield are annual data, we chose *λ = *100 for the analysis. We input the actual NCP winter wheat yield data to Eviews8, selected the Quick-Series Statistics-HP filter option under the data sequence menu, and used the output as the trend yield, *gt*. The actual yield, *Yt*, minus *gt* was used to obtain the winter wheat climatic yield, *ct*.

## Data Availability

The SAT (global), GPCP, CMAP, ER-SST V5, CPC Soil Moisture, GPH, *u* wind and v wind data all provided by the NOAA/OAR/ESRL PSD, Boulder, Colorado, USA, from their Web site at https://www.esrl.noaa.gov/psd/ are all publicly and freely available.
